# Oesophageal injury mimicking a tubular congenital oesophageal duplication—a diagnostic dilemma: a case report

**DOI:** 10.1093/bjrcr/uaae035

**Published:** 2024-10-03

**Authors:** Shreya Shrivastava, Priscilla Joshi, Shriyash Pinglikar

**Affiliations:** Department of Radiodiagnosis, Bharati Vidyapeeth (Deemed to be University), Pune, Maharashtra 411043, India; Department of Radiodiagnosis, Bharati Vidyapeeth (Deemed to be University), Pune, Maharashtra 411043, India; Department of Radiodiagnosis, Bharati Vidyapeeth (Deemed to be University), Pune, Maharashtra 411043, India

**Keywords:** newborn, intramural oesophageal dissection (IED), oesophageal injury, oesophageal duplication

## Abstract

Intramural oesophageal dissection (IED) is an uncommon condition in newborns marked by the separation of the mucosal and submucosal layers of the oesophageal wall, both transversely and longitudinally, which may or may not involve perforation. A neonate presented at 26 h of life with poor respiratory effort and lethargy. She was intubated and was put on mechanical ventilation. Radiograph of the neonate suggested malpositioned endotracheal tube. The fluoroscopic dye-study indicated gastroesophageal oesophageal reflux disease and nothing significant. On limited CT contrast study of thorax, a tubular structure was seen running just parallel to the oesophagus extending from the T2 to the T9 levels. Possibilities of a oesophageal duplication/IED were considered. The neonate underwent an endoscopy and gastrostomy on day of life (DOL) 9. On follow up at 3 months a repeat limited CT study was done with instillation of water-soluble contrast. The previously seen tubular structure running parallel to the oesophagus was no longer seen. This finding suggested a healed IED. This case report emphasizes the significance of multimodality imaging in the diagnosis of this condition.

## Introduction

Oesophageal injuries rarely occur in children, but they can be fatal, with mortality rate of up to 28% if they advance to perforation. Oesophageal injury typically occurs as result of iatrogenic interventions. Causes of oesophageal injury include gastric tube insertion, endoscopic dilatation, endotracheal intubation, and the insertion of a respiratory suction catheter.^1^ Iatrogenic manipulations can either lead to perforation or oesophageal dissection.

Intramural oesophageal dissection (IED) although a known entity, has rarely been seen in newborns. Diagnosis is achieved through contrast oesophagography, oesophagoscopy, or a combination of both. Nonoperative therapy has consistently demonstrated success.^2^

Gastrointestinal duplication occurs in 1/4500 live births, with roughly 20% of these cases involving oesophageal duplications.^3^ Approximately one third of all duplications originate from the foregut, which encompasses the oesophagus, stomach, and first and second part of the duodenum. Respiratory symptoms are frequently observed in foregut duplication, particularly when the bronchial tree is involved. In certain cases, the patients may exhibit respiratory distress and haemoptysis.^1^

Tubular variants are uncommon, typically communicating with the true oesophageal lumen and often sharing a significant length of the wall with the native oesophagus.^3^

After surgical excision of the additional oesophagus, prognosis is favourable in most cases.

## Case report

A late pre-term female baby born at 36 week 5 days of gestation was referred to our hospital in view of respiratory arrest, decreased level of consciousness, and decreased feed intake. Arterial blood gas (ABG) was suggestive of metabolic alkalosis with respiratory compensation. The neonate was delivered via lower segment caesarean section (LSCS), in view of thick meconium-stained liquor. The neonate cried immediately after birth.

As the feeding tube could not be passed beyond 16 cm, a possibility of trachea oesophageal fistula was entertained, and she was referred to the Department of Radiodiagnosis for a water-soluble upper gastrointestinal contrast study.

## Imaging findings

**Figure 1.**
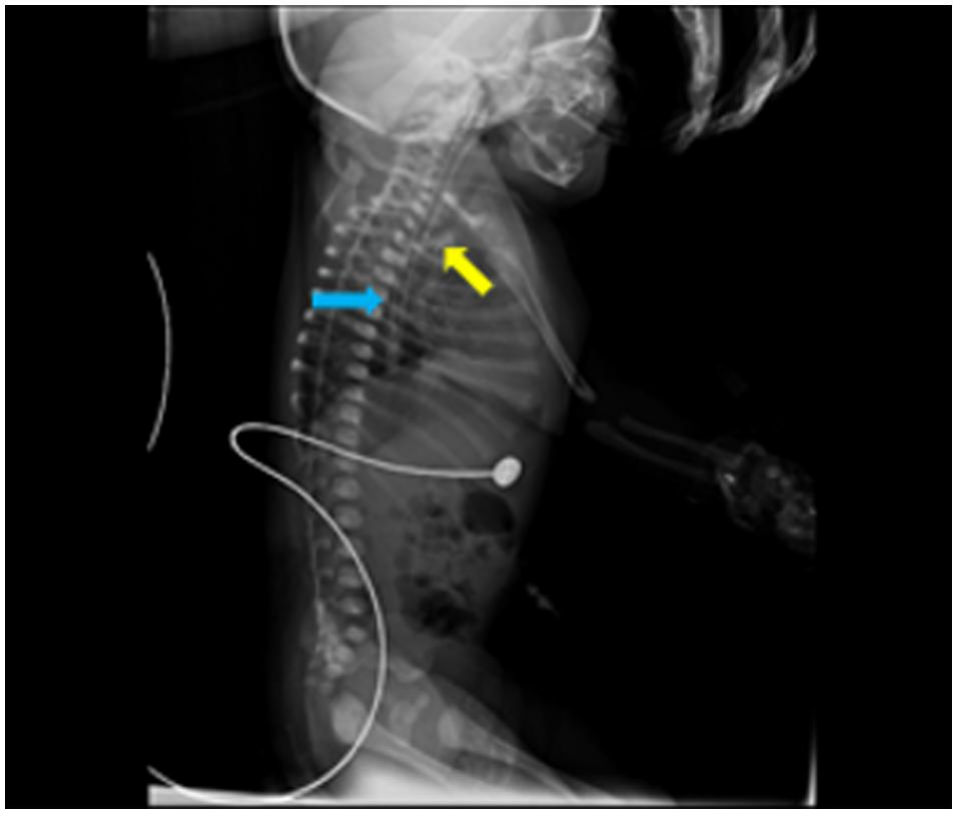
Lateral radiograph show malpositioned endotracheal tube (ETT) (blue arrow) in the oesophagus. A longitudinal translucent streak (yellow arrow) anterior to the oesophagus/malpositioned ETT denotes the air density in the trachea.

On the lateral chest radiograph, a wide bore tube was seen traversing through mouth and pharynx down to distal oesophagus, suggestive of a malpositioned endotracheal tube (ETT) (Figure 1).

**Figure 2.**
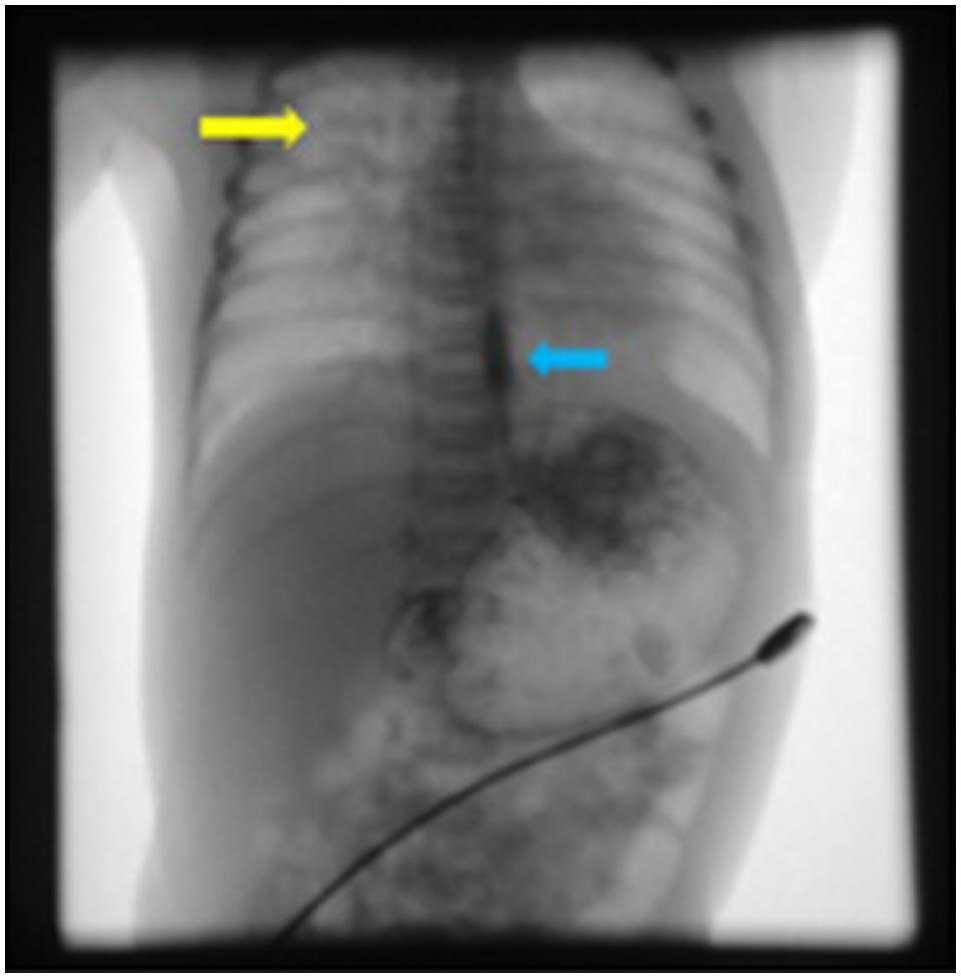
Under fluoroscopic guidance, frontal radiographs were obtained with oral contrast material spill from the laryngopharynx at the level of cricopharyngeus into the tracheo-bronchial tree (yellow arrow) and gastroesophageal reflux was observed in Trendelenburg position (blue arrow).

On contrast oesophagography, during the initial swallowing phase there was spillage of contrast from the laryngopharynx at the level of cricopharyngeus into the tracheo-bronchial tree (Figure 2).

Smooth unobstructed passage of contrast was noted from the oropharynx to the hypopharynx, oesophagus, and stomach.

Contrast was also given in prone position and no communication was seen between oesophagus and tracheo-bronchial tree. However, reflux of contrast was noted from the level of gastro-oesophageal junction into the thoracic oesophagus in Trendelenburg position (Figure 2).

**Figure 3.**
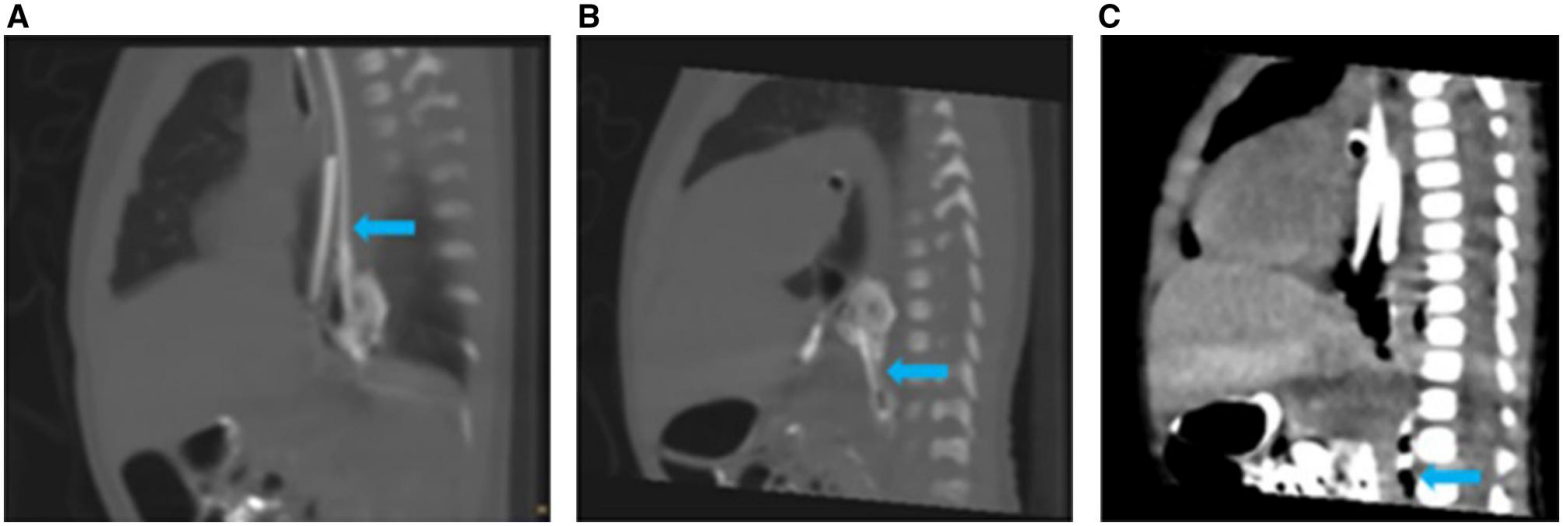
On limited CT study, sequential sagittal images show opacification of the second lumen (blue arrow), which is running parallel to the oesophagus (A). It is continuing in the posterior mediastinum into a pouch like structure (B) and ends as blind pouch, infra-diaphragmatically with few air foci in it (C).

After proper clinico-radiological evaluation, duplication of the oesophagus was suspected. A limited non contrast CT scan was done, which showed an oval air containing structure posterior to the oesophagus possibly due to a duplication. This structure could be traced cranially up to the T2 vertebral level and caudally up to the level of the renal hilum. With one feeding tube in situ in the stomach another feeding tube was directed posteriorly, while passing it into the proximal oesophagus. Contrast instilled into this appeared to opacify the second lumen.

Diluted non ionic iodinated contrast was instilled through the nasogastric tubes (NGTs), one with its tip in the stomach (tube-1) and the other in the blind end of the duplicated oesophagus (tube-2).

**Figure 4.**
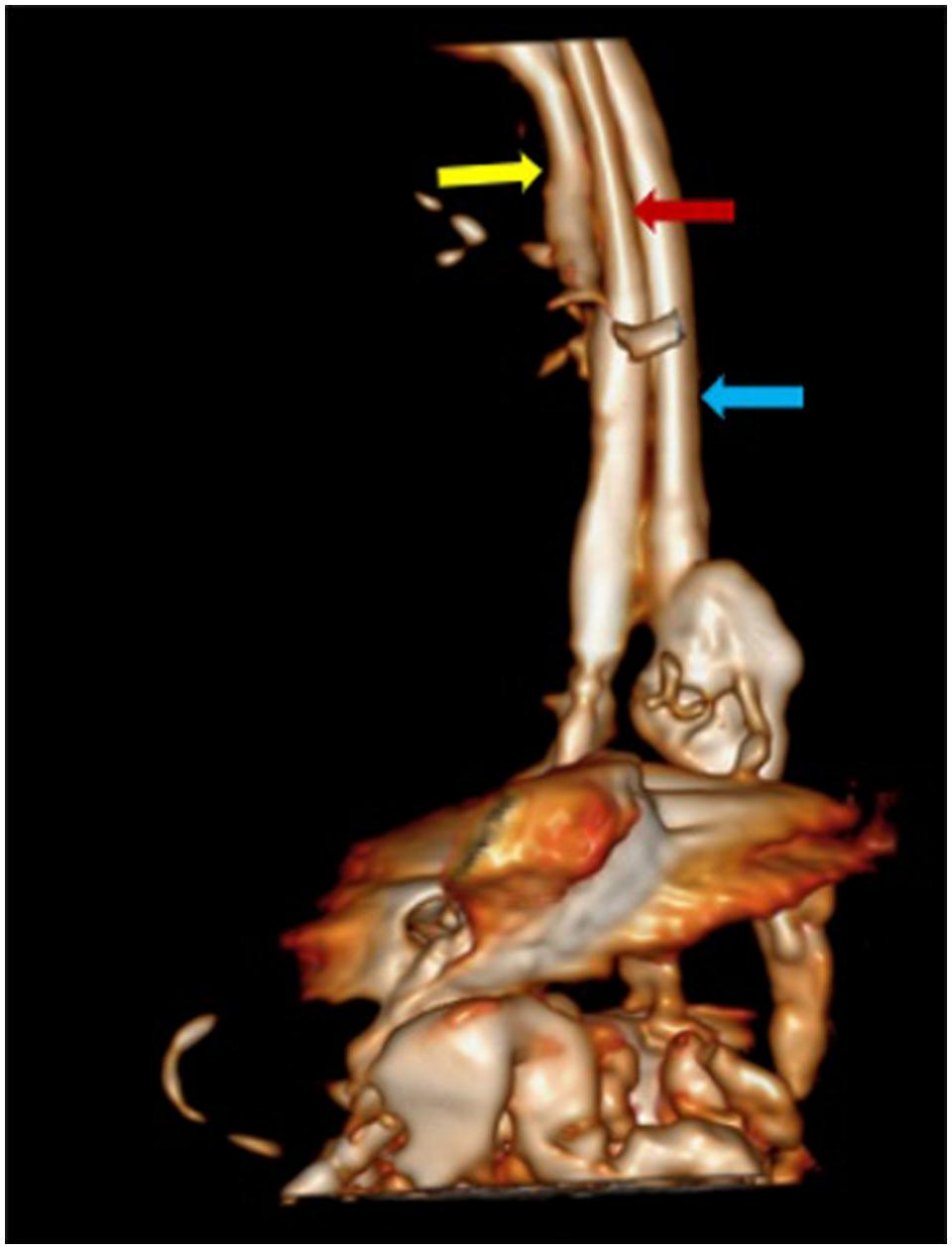
3D volume rendered image showing a blind ending duplicate lumen (blue arrow) running parallel to the oesophagus (red arrow). The yellow arrow represents the trachea anterior to the oesophagus.

After instillation of diluted non-ionic contrast in tube-2, the second lumen was seen arising from the oesophagus posteriorly at T2 vertebral level. This was seen running parallel to the oesophagus, continuing into a pouch-like structure at T8-T9 level which was in the posterior mediastinum and was air containing (Figure 3). A thin streak of contrast was seen continuing infra-diaphragmatically up to the level of the renal hila. This measured approximately 4.5 × 2.7 mm in axial dimensions and was air containing (Figure 4).

A possibility of oesophageal duplication with infra diaphragmatic continuation of the duplicated oesophagus was considered with a differential diagnosis of an iatrogenic false lumen.

No pneumomediastinum was seen on the chest radiographs.

No associated vertebral segmentation anomalies were seen.

On endoscopy the opening of the second lumen showed oedema at its periphery. The endoscopist did not enter the second lumen to avoid the injury.

Ultrasonography of the chest was not performed as it was not expected to reveal any additional findings.

Baby was taken for gastrostomy on day of life (DOL) 9. After observation and conservative management, the baby was discharged. Baby was hemodynamically stable on room air, tolerating gastrostomy feeds.

Radiological follow up was planned after 3 months to confirm presence and patency of the duplicate lumen.

Limited contrast CT study of the thorax was done at 3 months of age. The infant was asymptomatic and showed good weight gain.

A NGT was introduced into the stomach, the tube was withdrawn and positioned at T1-T2 vertebral level. Three to 4 cc of diluted contrast instilled through the tube and the patient was scanned.

**Figure 5.**
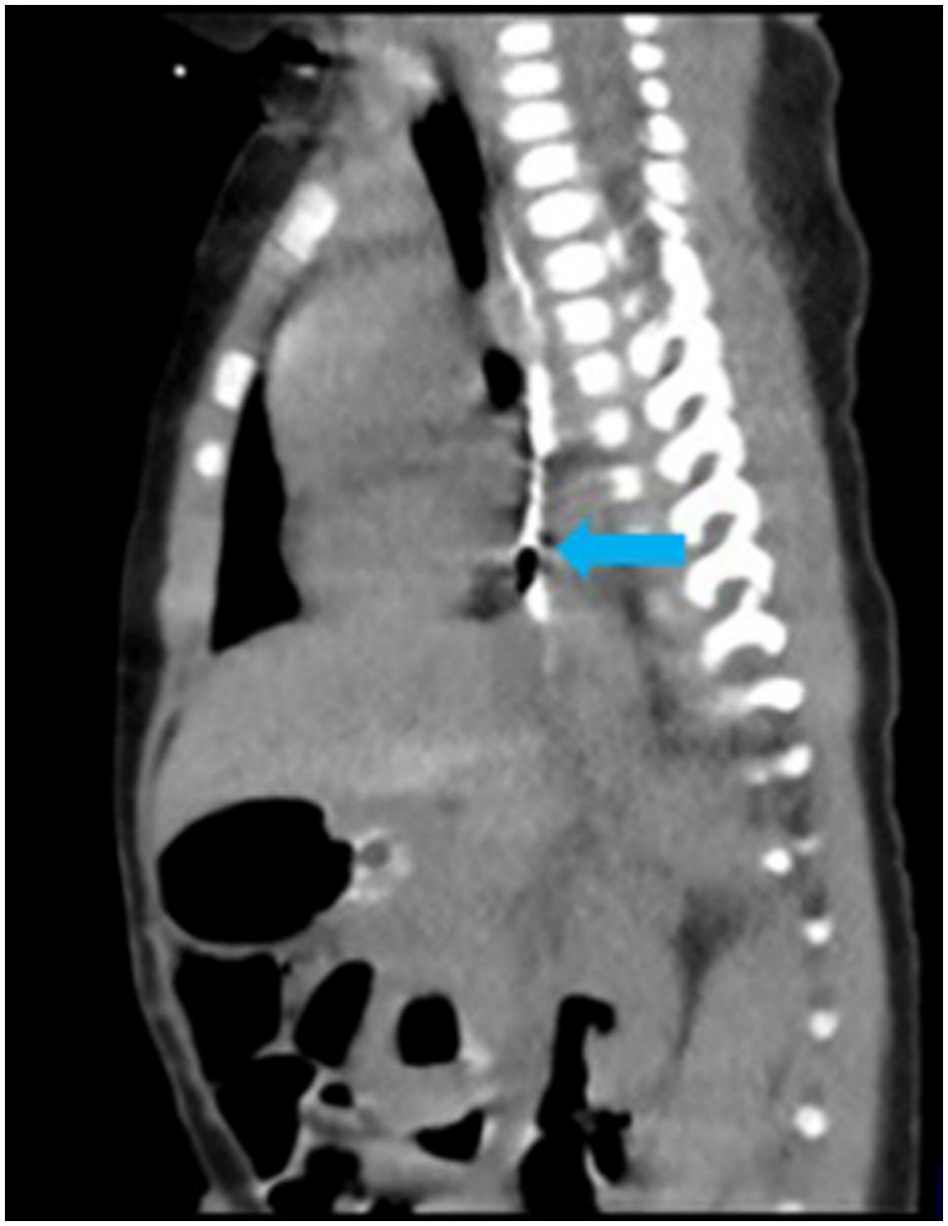
On limited contrast CT study—sagittal image showing oesophagus (arrow) with no other abnormal structure around it.

Smooth non obstructed passage of contrast was seen through the oesophagus into the stomach. Oesophagus and stomach appeared normal. No other tubular structure which was contrast or air filled could be seen in the thorax or abdomen. No extravasation of contrast was seen (Figure 5).

No tubular structure could be demonstrated running parallel to the oesophagus to suggest oesophageal duplication. Also, no fistulous communication or tract was seen.

These findings suggested the possibility of an IED which had healed.

## Discussion

IED is a rare condition that can occur either spontaneously or because of medical procedures. Iatrogenic causes include: (1) gastrointestinal endoscopy, (2) endotracheal intubation, and (3) NGT insertion.^4^

However, a differential diagnosis of oesophageal tubular duplication cyst which is also a rare congenital anomaly, needs to be considered.

Imaging findings in both cases will show a second tubular structure running alongside the oesophagus, making it difficult to reach a definite diagnosis.

IED is characterised by separation of mucosa from the submucosa. On the other hand, histopathological examination of duplication cyst reveals gastric-type mucosa with a well-developed submucosa, muscularis propria, and serosa.^5^

Management of the IED is conservative. It entails keeping the neonate on a nil-by-mouth status, nutritional support, providing fluid resuscitation, and administering broad-spectrum antibiotics.^5^

Alternate therapies under consideration include endoscopic interventions such as mucosal septum incision, balloon dilatation, and metal stent insertion.

Surgery may be indicated for patients who do not respond to conservative management, or who have complications such as oesophageal perforation, haemorrhage, or abscess formation.^5^

It is well documented that surgical excision is a viable option for the duplication of the oesophagus.^6^

In this case, the patient was conservatively managed for 3 months, after which imaging was planned to confirm the diagnosis. Radiological investigations after 3 months showed complete healing of the false lumen, which supported the diagnosis of an IED.

Correct position of NGTs or ETTs and any iatrogenic injury caused by them can be prevented by appropriate imaging, that is, a chest radiograph following introduction of the tube. Radiographic confirmation of NGTs/ETTs placement is commonly considered the gold standard, although it subjects patients to ionizing radiation. Studies have demonstrated that ultrasonography can aid in the placement of NGTs/ETTs in paediatric patients, helping to reduce the frequency of incorrect placements during insertion.^7^^,^^8^

Iatrogenic oesophageal perforation is uncommon in children but can occasionally occur in premature infants due to repeated intubation or NGT insertion. Prompt and precise diagnosis, followed by appropriate treatment, is crucial, as this condition can be fatal.^9^ Radiological investigations are helpful in ruling out complications such as pleural effusion, pneumomediastinum and emphysema and there-by aiding in treatment.

## Conclusion

Intramural oesophageal duplication in the new born has rarely been reported. It is important to be aware of this condition, investigate and follow up the condition appropriately.

Radiological investigations play a vital role in the diagnosis. As in reported case, recognition of this complication and appropriate management, helped in reaching the correct diagnosis. Non-ionic contrast was used when performing the study to prevent contrast related lung injury which could occur in case of aspiration or in the presence of a tracheooesophageal fistula.

## Learning points

- Intramural oesophageal dissection (IED), though rarely seen, must be considered in newborns presenting with respiratory distress who have undergone intervention. Care should be taken during intervention to avoid iatrogenic injury to newborn.
- Imaging including ultrasound and radiographs can assist in accurate placement of nasogastric tubes/endotracheal tubes in neonates and paediatric patients.
- Patients should be conservatively managed treatment when IED is suspected. Early intervention should not be resorted to, as this might lead to catastrophe.
